# Supplementary material for: Designing Aedes (Diptera: Culicidae) Mosquito Traps: The Evolution of the Male Aedes Sound Trap by Iterative Evaluation
Source: Insects. 2021 Apr 27;12(5):388. doi: 10.3390/insects12050388 (PMC8146609; doi:10.3390/insects12050388)
Supplement: Supplementary file 1 [file insects-12-00388-s001.zip › Figure S1.pdf]

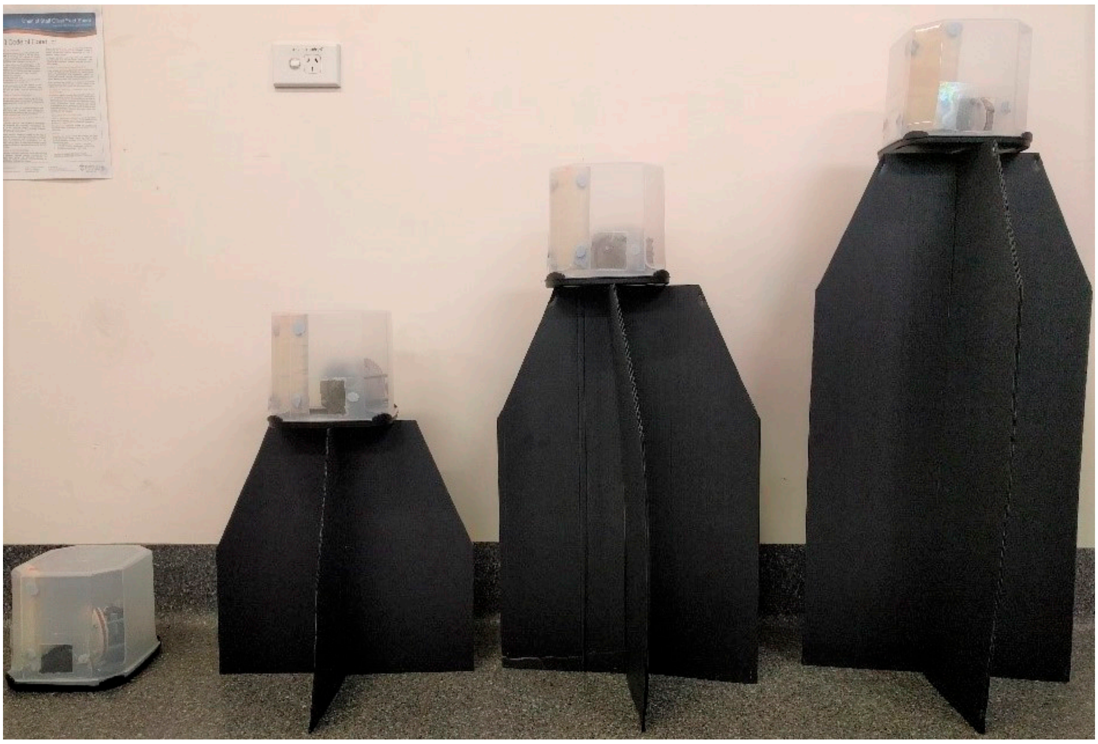

Figure S1. The various MAST base heights tested, 0 cm, 40 cm, 60 cm and 80 cm as shown in the figure from left to right.
