# Supplementary material for: Designing Aedes (Diptera: Culicidae) Mosquito Traps: The Evolution of the Male Aedes Sound Trap by Iterative Evaluation
Source: Insects. 2021 Apr 27;12(5):388. doi: 10.3390/insects12050388 (PMC8146609; doi:10.3390/insects12050388)
Supplement: Supplementary file 1 [file insects-12-00388-s001.zip › Figure S3.pdf]

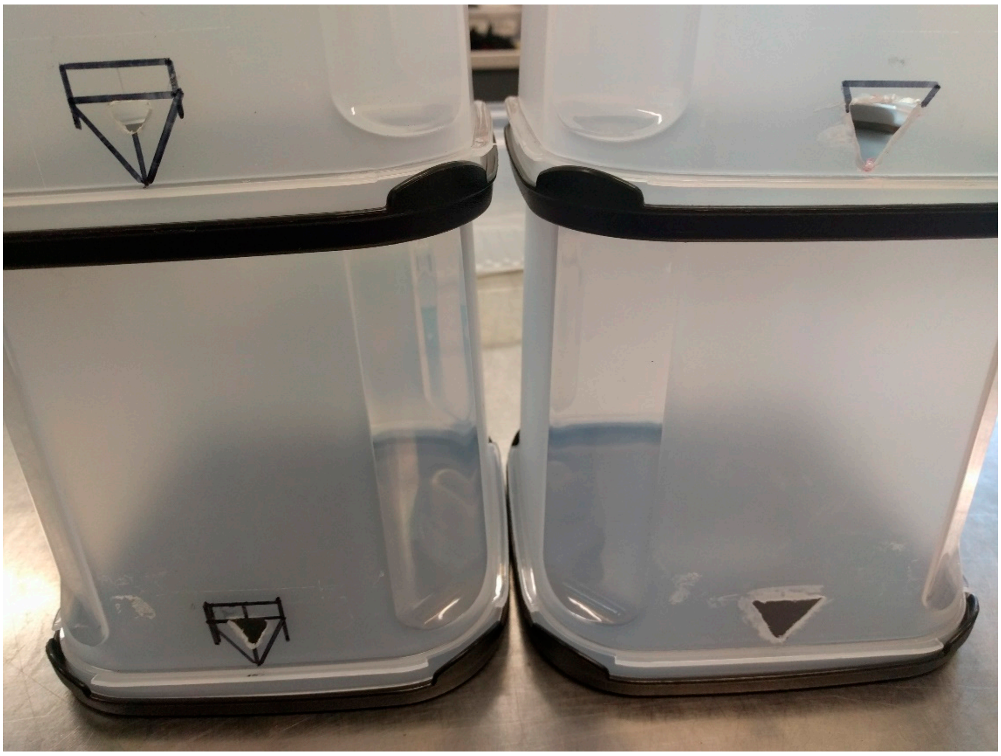

Figure S3. The various MAST entry sizes trialled top left (1 cm), bottom left (1.5 cm), top right (2 cm) and bottom right (2.5 cm). Black marks were removed before trials began.
