# Supplementary material for: Designing Aedes (Diptera: Culicidae) Mosquito Traps: The Evolution of the Male Aedes Sound Trap by Iterative Evaluation
Source: Insects. 2021 Apr 27;12(5):388. doi: 10.3390/insects12050388 (PMC8146609; doi:10.3390/insects12050388)
Supplement: Supplementary file 1 [file insects-12-00388-s001.zip › Figure S5.pdf]

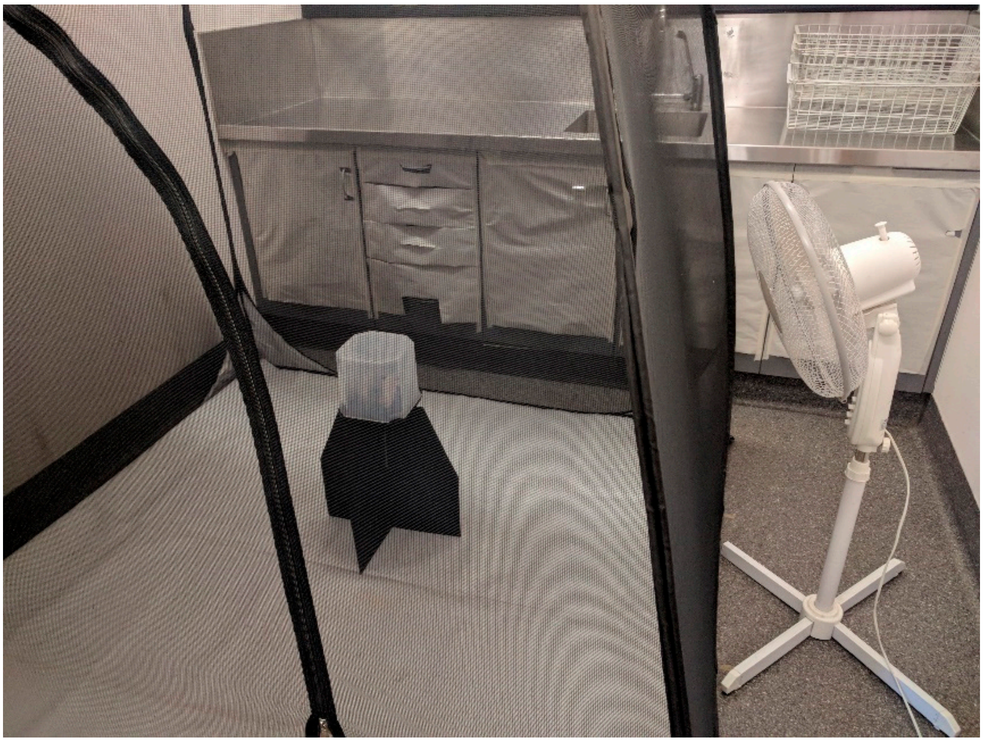

Figure S5. Experimental design to test the influence of wind on male *Ae. aegypti* capture rates in the MAST.
