# Supplementary material for: Designing Aedes (Diptera: Culicidae) Mosquito Traps: The Evolution of the Male Aedes Sound Trap by Iterative Evaluation
Source: Insects. 2021 Apr 27;12(5):388. doi: 10.3390/insects12050388 (PMC8146609; doi:10.3390/insects12050388)
Supplement: Supplementary file 1 [file insects-12-00388-s001.zip › Table_S1.pdf]

Table S1. Summary of trials performed

Trial	Aspect	Aim of research	Result	Facility	Base	Head	Entrance	Sound lure settings
1	Base	How do various MAST base heights influence male catches in the small flight cage?	• Males were not caught in the MAST without a base.<br>• Increasing base height improved catch rate of *Ae. aegypti* males.	Semi-field flight cage	No base or 40 cm, 60 cm or 80 cm high corflute	4 L translucent	1 square (5 cm sides)	500 Hz, 63 dB, intermittent (30 s)
2	Base	How do various MAST base heights influence male catches in a single premises in the field?	• Set simultaneously, on opposite sides of a house, no difference in male *Ae. aegypti* catches in MASTs when using bases 23 or 45 cm high.	Single premises	Black buckets either 25 or 45 cm high	2.5 L clear	1 triangle (2 cm sides)	500 Hz, 63 dB, intermittent (30 s)
3	Base	How do various MAST base heights influence male catches in multiple premisses in the field?	• From Latin squares run in CNS city, no difference in male *Ae. aegypti* catches in MASTs when using bases 27 or 45 cm high.	Multiple premisses	Black buckets either 27 or 45 cm high	2.5 L clear	1 triangle (2 cm sides)	500 Hz, 63 dB, intermittent (30 s)
4	Head	How does the number, size and shape of the entrance influence male *Ae. aegypti* catch rates in the MAST?	• Reducing the number and size of MAST entrances did not reduce the numbers of male *Ae. aegypti* caught within a 5 minute period.	Semi-field flight cage	Corflute 40 cm high	4 L translucent	2 squares (5 cm sides), 1 square (5 cm sides), 1 square (2.5 cm sides), 1 triangle (2.5 cm sides)	500 Hz, 63 dB, intermittent (30 s)
5	Head	How do various triangular entrance sizes influence male *Ae. aegypti* catch rates in the MAST?	• Each increase in size of the MAST triangular entrance hole significantly increased male *Ae. aegypti* catch rates within a 5 minute period.	Semi-field flight cage	Corflute 40 cm high	4 L translucent	1 triangle (2.5 cm sides), 1 triangle (2 cm sides), 1 triangle (1.5 cm sides), 1 triangle (1 cm sides)	500 Hz, 63 dB, intermittent (30 s)
6	Lure	How do continuous or intermittent tones influence male *Ae. aegypti* catch rates in the MAST?	• Changing the sound lure playback form continuous to 30 seconds on-off does not significantly reduce *Ae. aegypti* catches.	Semi-field flight cage	Corflute 40 cm high	4 L translucent	1 triangle (2.5 cm sides)	500 Hz, 63 dB, continuous or intermittent (30 s)
7	Lure	How do different volume settings influence male *Ae. aegypti* catch rates in the MAST?	• Changing the MAST volume settings didn't significantly influence male *Ae. aegypti* catch rates.	Semi-field flight cage	Corflute 40 cm high	4 L translucent	1 triangle (2.5 cm sides)	500 Hz, 63 dB, 68 dB or 74 dB,

								intermittent (30 s)
8a	Lure	How does setting the sound lure to 450 Hz or 500 Hz influence male *Ae. aegypti* catch rates in the MAST as males age?	• MASTs with sound lures set to 450 or 500 Hz displayed high catch rates of male *Ae. aegypti*.<br>• Male *Ae. aegypti* of varying ages were caught at comparably high rates in the MAST.	Tent	Black buckets 45 cm high	4 L translucent	1 triangle (2.5 cm sides)	450 Hz or 500 Hz, 63 dB, intermittent (30 s)
8b	Lure	How does setting the sound lure to 550 Hz, 600 Hz or off influence male *Ae. aegypti* catch rates in the MAST?	• Male *Ae. aegypti* were caught in high rates within MASTs with sound lures set to 550 or 600 Hz.<br>• No male *Ae. aegypti* were caught at in MASTs with sound lures set 0 Hz (lure turned off)	Tent	Black buckets 45 cm high	4 L translucent	1 triangle (2.5 cm sides)	0 Hz, 550 Hz or 600 Hz, 0 dB or 63 dB, intermittent (30 s)
9	Environment	How does wind blowing on the MAST influence male *Ae. aegypti* catch rates?	• Wind negatively affected trap catches of male *Ae. aegypti*.<br>• If the trap is exposed to a continuous wind ≤1.5 ms^{-1} then male *Ae. aegypti* did not enter it.<br>• However, they will still swarm in the leeward side of such a wind and, if the MAST is facing leeward, ~50% will enter it. Similarly, male *Ae. aegypti* will still enter the trap with an intermittent wind.	Tent	Corflute 40 cm high	4 L translucent	1 triangle (2.5 cm sides)	500 Hz, 63 dB, intermittent (30 s)
